# Image-guided intensity modulated radiotherapy with helical tomotherapy for postoperative treatment of high-risk oral cavity cancer

**DOI:** 10.1186/1471-2407-11-37

**Published:** 2011-01-27

**Authors:** Chen-Hsi Hsieh, Ying-Shiung Kuo, Li-Jen Liao, Kawang-Yu Hu, Shih-Chiang Lin, Le-Jung Wu, Yu-Chin Lin, Yu-Jen Chen, Li-Ying Wang, Yen-Ping Hsieh, Shoei Long Lin, Chun-Yi Chen, Chien-An Chen, Pei-Wei Shueng

**Affiliations:** 1 Department of Radiation Oncology, Far Eastern Memorial Hospital, Taipei, Taiwan; 2 Department of Dentistry and Oral Surgery, Far Eastern Memorial Hospital, Taipei, Taiwan; 3 Department of Otolaryngology, Far Eastern Memorial Hospital, Taipei, Taiwan; 4 Division of Medical Oncology and Hematology, Department of Internal Medicine, Far Eastern Memorial Hospital, Taipei, Taiwan; 5 Department of Radiation Oncology, Mackay Memorial Hospital, Taipei, Taiwan; 6 Department of Medical Research, Mackay Memorial Hospital, Taipei, Taiwan; 7 Institute of Traditional Medicine, School of Medicine, National Yang-Ming University, Taipei, Taiwan; 8 Graduate Institute of Sport Coaching Science, Chinese Culture University, Taipei, Taiwan; 9 School and Graduate Institute of Physical Therapy, College of Medicine, National Taiwan University, Taipei, Taiwan; 10 Department of Healthcare Administration, Asia University, Taichung, Taiwan; 11 Department of Radiation Oncology, National Defense Medical Center, Taipei, Taiwan; 12 Department of Surgery, Taipei Hospital, Department of Health, Taipei, Taiwan; 13 Division of Medical Oncology, Department of Internal Medicine, Taipei Hospital, Department of Health, Taipei, Taiwan; 14 Division of Traditional Chinese Medicine, Taipei Hospital, Department of Health, Taipei, Taiwan

## Abstract

**Background:**

The aim of this study was to assess the treatment results and toxicity profiles of helical tomotherapy (HT) for postoperative high-risk oral cavity cancer.

**Methods:**

From December 6, 2006 through October 9, 2009, 19 postoperative high-risk oral cavity cancer patients were enrolled. All of the patients received HT with (84%) or without (16%) chemotherapy.

**Results:**

The median follow-up time was 17 months. The 2-year overall survival, disease-free survival, locoregional control, and distant metastasis-free rates were 94%, 84%, 92%, and 94%, respectively. The package of overall treatment time > 13 wk, the interval between surgery and radiation ≤ 6 wk, and the overall treatment time of radiation ≤ 7 wk was 21%, 84%, and 79%, respectively. The percentage of grade 3 mucositis, dermatitis, and leucopenia was 42%, 5% and 5%, respectively.

**Conclusions:**

HT achieved encouraging clinical outcomes for postoperative high-risk oral cavity cancer patients with high compliance. A long-term follow-up study is needed to confirm these preliminary findings.

## Background

The location of the primary tumor site in head and neck cancer is an important prognostic factor [1-3]. Amdur *et al*. [1] reported that the primary site of head and neck tumors is significantly important for predicting disease control. In addition, Peters *et al*.[2] also reported that oral cavity primary tumors are one of the risk factors associated with progressively increased risk of recurrence. In another published report, the authors also found that oropharyngeal cancer patients had the greatest locoregional recurrence-free survival rate among oral cavity cancer (OCC) patients [3]. These reports suggest that the treatment for OCC is a challenge.

Besides prognostic factors [4,5] directly related to the tumor or the surgical specimen, treatment-related variables may also account for differences in clinical outcomes, including the total dose of radiation [2], package of overall treatment time (POTT) [6] and the overall treatment time of radiation therapy (OTTRT) [7]. In a prospective randomized study, a significantly higher locoregional recurrence rate was found among patients who received a dose of < 54 Gy compared with those who received a dose of > 57.6 Gy [2]. An interval between surgery and radiotherapy prolonged than 7 wks was associated with a significant reduction in locoregional control [6]. In addition, shorter OTTRT was associated with better overall survival rate [7].

Helical tomotherapy (HT) is an image-guided new CT-based rotational intensity modulated radiotherapy (IMRT) that delivers highly conformal dose distributions to the target tissue [8]. Thus, this complex rotational treatment method has the ability to spare critical organs exposure to unnecessary radiation. The preliminary studies of HT for locally advanced oropharyngeal cancer achieved encouraging results [9].

We also noted these encouraging results for oropharyngeal tumors treated with HT. In addition, HT reduced the incidence of side effects during treatment that could made treatment without interruption possible. Because OCC is associated with progressively increased risk of recurrence, the clinicians are concerned about how radiation dosage, POTT, and OTTRT affect treatment results. Therefore, we report our initial clinical experience with postoperative OCC patients treated with HT, focusing on the clinical outcome and toxicity.

## Methods

### Patient characteristics

From December 6, 2006 through October 9, 2009, we identified 19 patients with locally advanced OCC who had undergone surgery followed by postoperative HT (POHT) with or without chemotherapy at Far Eastern Memorial Hospital. Retrospective patient data was collected with the approval of the Institutional Review Board of Far Eastern Memorial Hospital. Staging investigations included complete history and physical examination, fiber optic endoscopic evaluation, complete blood counts, liver function tests, chest X-ray, magnetic resonance imaging (MRI) of the head and neck region, which was done before surgery, and a dental evaluation. Bone scans, computed tomography (CT) of the chest and abdomen were obtained whenever possible before the beginning of treatment. The disease was staged according to the American Joint Committee on Cancer Staging Classifications 6^th ^edition, which is based on the pathological findings after radical surgery.

### Radiation therapy

A type-S thermoplastic head frame (MT-CHFN-C, Civco MedTec, Kalona, Iowa, USA) were used for head and shoulder immobilization. CT with a 3-mm slice thickness was done for treatment planning. Target objects and normal structures were contoured using the Pinnacle 3 Treatment Planning System (Philips Healthcare, Madison, Wisconsin, USA). The preoperative MRI images were retrieved on a Pinnacle workstation and fused with the CT images for contouring and preoperatively confirming the location of the gross tumor and postoperative flap in all patients.

### Delineation of target volumes

The determination of clinical target volumes (CTVs) was based on the incidence and location of metastatic neck nodes from various head and neck subsides [10]. The CTV1, using preoperative MRI fused with the CT images to confirm the location of the gross tumor and the postoperative flap, was defined as encompassing the preoperative gross tumor and postoperative flap plus a 0.8- to 1-cm margin, which included the resection bed with soft-tissue invasion by the tumor or extracapsular extension (ECE) by metastatic neck nodes truncating air, and uninvolved bones. CTV2 was defined as a high-risk subclinical area primarily including the pathologically uninvolved cervical lymph nodes, deemed as elective nodal regions, or prophylactically treated neck areas [3,10,11]. CTV3 was designated as the low-risk area of potential subclinical disease. To account for organ motion and patient setup errors, the planning target volumes (PTVs) encompassed the CTVs plus a margin 3 mm. CTV1 received 60-66 Gy in 30-33 fractions; 64-66 Gy was delivered to high-risk OCC patients and 60 Gy was delivered to intermediate-risk OCC patients. For CTV2, 59.4-60 Gy/30-33 fractions was delivered and for CTV3, 51.2-54 Gy/30-33 fractions was delivered. The dose constraints for organs at risk (OARs) were as follows: (1) brainstem: maximum dose, 54 Gy; (2) spinal cord: maximum dose, 45 Gy; (3) optic chiasm and optic nerve: maximum dose, 45 Gy; (4) bilateral parotid glands: mean dose, < 30 Gy, median dose, < 26 Gy, and whole parotid gland volume with < 20 Gy that larger than 20 cc; (5) 2/3 of glottic larynx < 50 Gy; and (6) inner ear: mean dose, < 50 Gy; (7) mandible: maximum dose: 70 Gy.

The field width, pitch, and modulation factor (MF) used for treatment planning optimization were 2.5 cm, 0.32, and 3.0, respectively. Maximum importance was given to target dose coverage. The constraints on dose and penalty were adjusted accordingly during optimization. All patients underwent daily megavoltage CT for setup verification [12].

### Dose-volume analysis of treatment plans

Dose-volume histograms of the PTVs and the critical normal structures were analyzed. No more than 20% of the PTV received more than 110% of its prescribed doses, and no more than 1% of any PTV received less than 93% of its prescribed doses. For the critical organs with functional subunits organized in a series such as the brainstem, spinal cord, and cochlea, the maximum point dose was examined. For critical organs with functional subunits organized in parallel such as the parotids (i.e., entire gland including deep and superficial lobes), the median dose was examined.

### Chemotherapy

Sixteen patients received concurrent chemotherapy. Three patients did not receive chemotherapy concurrently with radiotherapy because they refused concurrent therapies. During radiotherapy, the patients who received chemotherapy were treated with cisplatin (30 mg/m^2^) plus fluorouracil (5-FU, 425 mg/m^2^) and leucovorin (30 mg/m^2^), both intravenously each week.

### Follow-up

All patients were evaluated at least once a week during radiotherapy. At the completion of radiation, patients were then evaluated every 3 months for the first 2 years. At each follow-up visit, a physical examination, including a fiber-optic endoscopic examination and palpation of the neck was performed. Post-treatment MRI of the oral cavity and neck was done 1, 3, and 6 months after completion of radiotherapy. Acute toxicities (occurring < 90 days after beginning radiotherapy) and late toxicities (occurring > 90 days after beginning radiotherapy) were defined and graded according to the Common Terminology Criteria for Adverse Events v3.0 (CTCAE v3.0). The earliest date of detecting Grade 3 or worse toxicity was recorded.

### Statistical methods

Descriptive statistics (mean, median, proportions) were calculated to characterize the patient, disease, and treatment features as well as toxicities after treatment. The overall survival (OS), disease-free survival (DFS), locoregional progression-free (LRPF), and distant metastases-free (DMF) rates were estimated using the Kaplan-Meier product-limit method [13]. Freedom from local progression was defined as the absence of the primary tumor on physical examination and on any radiographic examination (CT and MRI). Durations were calculated from the date of pathologic proof. All analyses were performed using the SPSS, version 12.0 (SPSS, Chicago, Illinois, USA).

## Results

### Patient characteristics

Seventeen men and 2 women were included in the study. They had a median age of 50 years (range, 24-70 years). The subsites of the tumors were located in the oral tongue (47%) and buccal mucosa (32%). Of the 19 patients, 53% had positive or close surgical margins, while 42% were ECE (+). The disease stage distribution included Stage II (1/19, 5%), Stage III and IVa (18/19, 95%). Almost all (90%) of the patients had more than two risk factors. (Table 1)

**Table 1:** Patient characteristics

	Tomotherapy (N = 19)
	
Variable	No. of patient (%)
**Age **(years)	
Median	50
Range	24-70
**Gender**	
Male	17 (89.5%)
Female	2 (10.5%)
**Smoking**	
Yes	16 (84.2%)
No	3 (15.8%)
**Alcohol drinking**	
Yes	10 (52.6%)
No	9 (47.4%)
**Betel nut chewing**	
Yes	12 (61.2%)
No	7 (36.8%)
**^ECOG Performance Status**	
0	9 (47.4%)
1	9 (47.4%)
2	1 (5.2%)
**Subsite**	
Oral tongue	9 (47.4%)
Buccal mucosa	6 (31.6%)
Gingiva	3 (15.8%)
Retromolar trigone	1 (5.3%)
**Pathology**	
Squamous cell carcinoma	19 (100%)
**Resection-margin status**	
Positive	1 (5.3%)
Close	9 (47.4%)
Negative	9 (47.4%)
**Extracapsular spread**	
Positive	8 (42.1%)
Negative	11 (57.9%)
**Perineural involement**	
Positive	14 (73.7%)
Negative	5 (26.3%)
**Lymphovascular Space Involvement**	
Positive	13 (68.4%)
Negative	6 (31.6%)
**Lymph-node involvement **≥**2 Positive**	
Positive	11 (57.9%)
Negative	8 (42.1%)
**Pathology stage**:	
**Tumor stage**	
Stage II	1 (5.3%)
Stage III	5 (26.3%)
Stage IVA	13 (68.4%)
Stage IVB	0
**Primary Tumor stage**	
T1	0
T2	6 (31.6%)
T3	8 (42.1%)
T4a	5 (26.3%)
T4b	0
**Regional Lymph Node stage**	
N0	5 (26.3%)
N1	3 (15.8%)
N2a	0
N2b	8 (42.1%)
N2c	3 (15.8%)
N3	0

*Abbreviations:*

ECOG Performance Status = Eastern Cooperative Oncology Group Performance Status.

### Treatment outcomes

**Figure 1:**
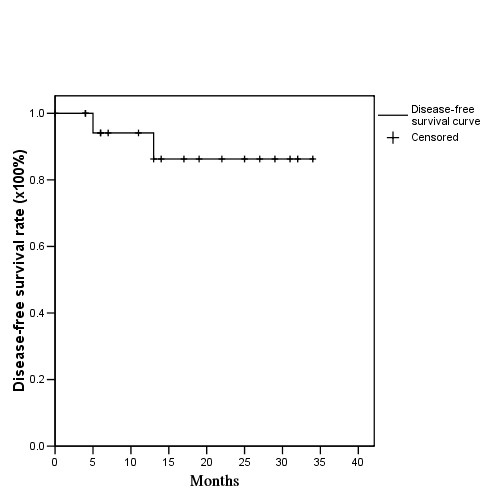
**Actuarial 2-year disease-free survival rates for postoperative oral cavity cancer patients treated with postoperative helical tomotherapy, with or without concurrent chemotherapy**.

The median and mean follow-up time was 17 months and 31 ± 2 months (range, 4-34 months). The 2-year actuarial OS, DFS, LRPF, and DMF rates were 94%, 84%, 92%, and 94%, respectively. (Figure 1 and Table 2) One patient had disease progression with lung metastasis and another had involve-field failure after CCRT 6 and 13 months, respectively.

**Table 2:** The 2-year estimated overall survival (OS), disease-free survival (DFS), locoregional progress-free survival (LRPF) and distant metastasis-free (DMF) rate of postoperative irradiation with or without of chemotherapy for high-risk oral cavity cancer at the Far Eastern Memorial Hospital (FEMH) compared with selected published series

Selected published Series	No. of postoperative patient	Proportion of OCC	T3 -4	LN involvement ≥2 Positive	Stage III, IV	Resection margin positive or close	ECE	PNI	LVSI/or VEs	Mod- Ality	2-year			
		(%)	(%)	(%)	(%)	(%)	(%)	(%)	(%)	(%)	OS (%)	DFS (%)	LR PF (%)	DMF (%)
Eisbruch et al.[3]	133	20	-	-	91	7	48	-	-	OP→ IMRT +C/T	-	-	65¶	-
Studer et al.[15]	28	100	32	57	68	-	-	-	-	OP→ IMRT +/-C/T	83	87	91	95
Chao KS et al.[16]	65	17.6	52	56	86	-	-	-	-	OP→ IMRT	-	-	90	-
Yao M et al.[18]	55	100	56	36	91					OP→ IMRT	68	74	82	89
Gomez DR et al.[19]	35	100	40	38	80	43	36	54	26	OP→ IMRT +/-C/T	74	70	84	85
Chen WC et al., Taiwan[17]	22	100	-	32	100	5	32	-	-	OP→ IMRT +/-C/T	72	64	-	-
FEMH, Taiwan	19	100	68	58	95	52.6	42.1	73.7	68.4	OP→ HT +/-C/T	94	84	92	94

*Value estimated from survival curves. †High-risk OCC: extracapsular spread, Resection-margin positive or close, or more than one adverse factor. ¶ The estimated 2-year LRPF rate belong to OCC only.

Abbreviations:

OCC = oral cavity cancer; LN = Lymph node; ECE = Extracapsular extension; PNI = Perineural involvement; LVSI = Lymphovascular space involvement; VEs = vascular embolisms; OS = overall survival; DFS = disease-free survival; LRPF= locoregional progress-free survival; DMF = distant metastasis-free; OP = operation; RT = External beam radiation therapy; IMRT = intensity-modulated radiation therapy; HT = helical tomotherapy; C/T = chemotherapy; FEMH = Far Eastern Memorial Hospital.

### Compliance with and delivery of treatment

All patients received rotational, multiple-angle beam HT. More than 80% (16/19) of the patients received adjuvant concurrent chemoradiation therapy (CCRT). The median dose of radiation was 66 Gy (range: 60-66 Gy). The POTT (means from surgery to CCRT or POHT last day) > 13 weeks was only 21%. The interval between operation and CCRT or postoperative radiotherapy (IBOR) ≤ 6 wk *vs*. > 6 wk was 84% *vs*. 16%. The overall treatment time of radiotherapy (OTTRT) ≤ 7 wk *vs*. > 8 wk was 79% *vs*. 21%. (Table 3)

**Table 3:** The package of overall treatment time (POTT), interval between operation and post-operation radiotherapy (IBOR) and overall treatment time of radiation therapy (OTTRT) at the Far Eastern Memorial Hospital (FEMH) compared with selected published series

Selected published studies	Factors		Percentage	Locoregional control			Disease-free survival			Overall survival		
				
			(%)	2-year	3-year	5-year	2-year	3-year	5-year	2-year	3-year	5-year
**University of Texas M. D. Anderson Cancer Center, USA **[6]	POTT	<11 wks	64.2%	81%	78%	76%				64%	58%	48%
		>13 wks	10%	38%	38%	38%				25%	25%	25%
	IBOR	≤6 wks	54.7%	78%	78%	75%				68%	58%	48%
		>6 wks	45.3%	58%	50%	48%				50%	32%	25%
**VU University Medical Center, Netherlands **[7]	POTT	<11 wks	12%		86%							
		>13 wks	45%		71%							
	OTTRT	≤7 wks	31%	90-82%	85-75%		78-58%	72-49%		82-65%	74-55%	
		>8 wks	16%	56%	51%		42%	38%		53%	50%	
**University of Florida, USA **[20]	POTT	≤101 days	63%	70%	70%	70%						
		>101 days	17%	50%	40%	30%						
	IBOR	≤51 days	57%	72%	72%	72%						
		>51 days	17%	56%	50%	40%						
**University of Florida College of Medicine, USA **[22]	POTT	<100 days		60%								
		>100 days		14%								
**University of Texas M. D. Anderson Cancer Center, USA **[2]	IBOR	≤6 wks		77%								
		>6 wks		64%								
**University Hospital, Avda, Spain **[21]	POTT	≤150 days				77%						
		>150 days				63%						
	IBOR	≤ 50 days				83%						
		>50days				68%						
	OTTRT	≤ 60 days				75%						
		>60 days				68%						
**FEMH, Taiwan**	POTT	≤11 wks	42.1%	92%			84%			94%		
		>13 wks	21.1%									
	IBOR	≤6 wks	84.2%									
		>6 wks	15.8%									
	OTTRT	≤ 7 wks	78.9%									
		> 8 wks	5.2%									

Abbreviation:

POTT = Package of overall treatment time; IBOR = Interval between operation and post-operation radiotherapy; OTTRT = Overall treatment time of radiation.

### Dose-volume analysis

The average of D93% and V110% for the PTVs was 65.0 ± 2.7 Gy and 0.14 ± 0.34%, respectively. The mean of the median doses for both sides of the parotid glands was 25.7 Gy (right side: 19.4-39.0 Gy; left side: 11.5-51.6 Gy). The averages of the mean doses for the right and left side parotid glands were 29.6 Gy and 30.1 Gy, respectively. The means of the maximal doses for the spinal cord and brain stem were 34.7 ± 5.7 Gy (range: 23.3-44.5 Gy) and 28.2 ± 6.4 Gy (range: 8.9-36.1 Gy), respectively. The mean dose for the larynx was 30.6 ± 6.2 Gy (range: 24.3-43.9 Gy).

### Toxicities

The acute toxicities of POHT with or without chemotherapy are detailed in Table 4. No grade 3 of acute toxicity for xerostomia, anemia, thrombocytopenia, or body weight loss was noted during CCRT or POHT. Of the 19 patients, (5%, 1/19) had grade 3 dermatitis, 5% (1/19) leucopenia, and 42% (8/19) had grade 3 mucositis during treatment. For acute toxicity, 9 of 19 patients had grade 2 xerostomia, while the others had grade 1 xerostomia. On follow up, all patients recovered to grade 1 xerostomia.

**Table 4:** The selected published series on acute toxicity rate for postoperative external beam radiation therapy/intensity-modulated radiation therapy/helical tomotherapy for locally advanced head and neck cancer and/or oral cavity cancer patients

Institute	Treatment (percentage of using chemotherapy)	Fistula formation/or skin dehiscence	*≥ Grade 3						
			
			dermatitis	mucosistis	Body weight loss	^Xerostomia (acute)	Anemia	Leukopenia	Thrombocytopenia
RTOG 9501[4]	OP+RT+C/T	-	8%	30%	-	2%		38% (all of hematologic effects)	
EORTC 22931[5]	OP+RT+C/T	-	-	41%	-	14%	-	16%	-
RTOG 0024[14]	OP+RT+C/T	11%	29%	60%	-	-		12% (all of hematologic effects)	
Yu et al.[23]	OP+RT	8-29%	-	-	-	-	-	-	-
Jeremic et al.[24]	RT+C/T	-	-	-	29%	-	-	-	-
Capuano et al.[25]	RT+C/T	-	-	-	17%	-	-	-	-
Gomez et al.[19]	OP+IMRT +C/T (29%)	-	3%	23%	-	0%	-	-	-
Chen WC et al. Taiwan [17]	OP+IMRT +C/T (9%)	-	0	14%	-	-	-	-	-
FEMH, Taiwan	OP+HT +C/T (84%)	11%	5%	42%	`0%	`0%	0%	5%	0%

^Toxicity of xelostomia (Acute): Acute toxicities is defined as occurring < 90 days after beginning RT.

*The grade of toxicity is according to the Common Terminology Criteria for Adverse Events v3.0 (CTCAE v3.0).

Abbreviations:

OP = operation; RT = External beam radiation therapy; IMRT = intensity-modulated radiation therapy; HT = helical tomotherapy; C/T chemotherapy; FEMH = Far Eastern Memorial Hospital.

## Discussion

The previous reports of locally advanced head and neck cancer patients who underwent surgery followed by RT concurrent with or without chemotherapy had 2-year estimated OS, DFS, and LRPFS of 63%-83%, 58%-87%, and 65%-91%, respectively [3-5,14-19]. The 2-year actuarial OS, DFS, LRPFS, and DMF rates in the current study are 94%, 84%, 92%, and 94%, respectively. These results are compatible with the previous reports, suggesting HT is a feasible treatment for high-risk postoperative OCC patients. (Table 2)

The patients with ECE (+), close or positive surgical margins, and two or more other adverse features were categorized as the high-risk group. All patients in the current study are in the high-risk group. (Table 1) A Dutch group compared the intermediate-risk group and the high-risk group and found that the 3-year LRCs for OCC were 87% and 66%, respectively (p = 0.0005) [7]. In a report from the University of Florida, the 5-year LRC rate was 63% for those in the unfavorable group [20]. University of Texas M. D. Anderson Cancer Center also noted the 5-year actuarial LRC was 42% for the high-risk group [6]. The previous reports point out the LRC rate for the high-risk group of head and neck cancer ranges from 42%-66% [6,7,20]. POHT resulted in a LRC rate of 92% in the current study. The results support the feasibility of HT for postoperative OCC treatment.

IMRT offers excellent outcomes for LRC and OS in postoperative head and neck cancers [3,15-19]. (Table 2) Nevertheless, the LRC for OCC is lower than for other subsites of head and neck cancer, even when treated with IMRT [3]. Hinermen *et al*. [20] indicated that the LRC of T3/T4 and stage III/IV was worse than early T and early stages, respectively. Although Studer *et al*. [15] showed excellent outcomes for postoperative OCC treated with IMRT, the proportion of T3/T4 and stage III/IV in their study *vs*. ours is 32% *vs*.68% and 68% *vs*.95%, respectively. Because ECE (+) data and positive operating margins were not shown in the Studer study, we compared the proportions of T3/T4 and stage III/IV to explain the potential benefits of local control by HT for postoperative OCC. Gomez *et al*. [19] also provided impressive results for postoperative OCC treated with IMRT. The rates of LRC, T4, ECE (+) and positive operating margins in their study were 84%, 31%, 36%, and 43%, respectively. In the current study, the corresponding rates were 92%, 26%, 42%, and 53%, respectively. HT provided potential benefits for local control of postoperative OCC patients with high risk factors. Nonetheless, the probable reasons for these benefits could be either too short follow-up or the addition of chemotherapy to the radiotherapy regimen. Additionally, the image-guidance function of HT provided high quality and adaptive treatments such as planned rescanning and recontouring of the tumor target.

The other factors contributing to LRC and OS are dose, POTT, IBOR, and OTTRT. In a randomized study at M. D. Anderson Cancer Center, a significantly higher locoregional recurrence rate was found among patients who received doses of < 54 Gy; those who had significantly higher locoregional control rates received doses ≥ 63 Gy [2]. Patients with advanced head and neck cancer who were at high or intermediate risk of developing locoregional recurrences from various sites who received 63 Gy over the course of 7 wk *vs*. 63 Gy over 5 wk achieved locoregional control with the accelerated radiotherapy approximately 15% greater than those treated with conventional radiotherapy techniques. Moreover, OTTRT > 8 wk was the most important prognostic factor both in the high-risk and intermediate-risk patient groups [7]. These studies indicated a 6%-7.8% improvement of locoregional control with every week of shortening of the overall treatment time. For patients treated by conventional radiation techniques, Langendijk *et al*. [7] reported that the OTTRT was ≤ 8 wk in 52% of patients and the OTTRT was > 8 wk in 16% o patients. Muriel *et al*. [21] also reported similar results for postoperative irradiation times ≤ 55 days (39% of patients) and > 56 days (61% of patients). In contrast, 95% of our patients had OTTRT ≤ 8 wk, while only 5% had OTTRT > 8 wk in our study. Our data suggests that POHT could lead to improvements in OTTRT in comparison with other modalities. The IBOR is recognized as an important end point in some studies [6,20,22]. The results of the studies summarized in Table 3 indicate the importance of POTT, IBOR, and OTTRT. In the current study, most of the patients who received POHT completed the treatment course during the recommended intervals (POTT > 13 wk, IBOR ≤ 6 wk, and OTTRT ≤ 7 wk: 21%, 84% and 79% of patients, respectively) with a median high dose of 66 Gy. Thus, HT used in the adjuvant setting for postoperative OCC can result in high compliance rates that offer encouraging results.

The rates of fistula are reported to range from 8% to 29% in patients treated with surgery and postoperative radiotherapy (PORT) [23]. (Table 4) The fistula formation rate among our patients was 11%; this result reflects the fact that HT did not increase the rate of fistula formation even under the median high dose treatment, with or without concurrent chemotherapy. Grade 3 dermatitis occurred with PORT concurrent with chemotherapy at a rate of 3%-29% [4,14,19]; the corresponding rate in the current study was 5%. (Table 4) In the report by Gomez *et al*., a similar rate for grade 3 dermatitis was noted. Additionally, grades 1 and 2 dermatitis occurred in 40% and 51% of patients, respectively [19]. In the current study, grades 1 and 2 dermatitis occurred in 58% and 37% of patients, respectively. Patients had fewer episodes of grade 3 dermatitis than grades 1 or 2 with POHT. Less severe dermatitis appeared to occur with POHT. Chen *et al*. [17] reported no grade 3 dermatitis in their IMRT experience with postoperative OCC. Nonetheless, the addition of concurrent chemotherapy to PORT significantly increases severe adverse effects [4,5]. The proportion of CCRT in the study by Chen *et al*. was 9%, but in the current study, it was 84%. A similar reason for lower rate of mucositis reported by Chen *et al*. (14%) [17] and Gomez *et al*. (23%) [19] could be related to the lower proportion of patients who received concurrent chemotherapy. The incidence of body weight loss greater than 20% of the pre-diagnosis weight among patients with head and neck cancer undergoing CCRT ranges from 17%-29% [24,25]. In the current study, no grade 3 body weight loss was noted. (Table 4) Moreover, the incidence of grade 3 hematologic toxicities for postoperative OCC patients who received CCRT was 12%-38% [4,5,14]. In the current study, grade 3 leucopenia occurred in only 5% of patients, which could have been related to the different chemotherapy regimens. (Table 4) Grade 3 xerostomia was reported in 2% to 14% of patients on concurrent PORT and chemotherapy [4,5]. None of our patients had grade 3 xerostomia. (Table 4) When the mean parotid radiation dose can be kept to 26 Gy or less, both objective and subjective post-treatment improvement in salivary function occurs [26]. In the current study, the contours of the parotid glands were never changed from the true volume to obtain better dose distributions. Additionally, the optimization parameters could be loosened if concerns were present regarding adequate tumor coverage. The average of the median doses for both sides of parotid glands was 25.7 Gy, while the average mean dose for the right side parotid glands was 29.6 Gy, and that for the left side parotid glands was 30.1 Gy. In the current study, the late toxicity for xerostomia was all grade 1, which shows that POHT has the potential to provide better quality of life, when given as a definitive treatment for oropharyngeal cancer [9].

There are some limitations to our current study. First, the small case number and the retrospective study design make drawing statistical conclusions difficult, and no conclusions about recurrence in comparison with other modalities can be made. Second, the follow-up time is short so that late effects are insufficiently addressed. Third, not all patients who received postoperative CCRT that caused treatment results belonged to the pure experience of postoperative high-risk OCC were treated with POHT with concurrent chemotherapy.

## Conclusions

In this retrospective study, high-risk OCC patients receiving POHT completed the treatment course during the recommended interval with a high rate of compliance. Treatment toxicity was acceptable even in the setting of concurrent chemotherapy. Long-term follow-up is needed to confirm these preliminary findings.

## Competing interests

We have no personal or financial conflicts of interest and have not entered into any agreement that could interfere with our access to the research data or our ability to analyze the data independently, prepare the manuscript, and publish it.

## Authors' contributions

All authors read and approved the final manuscript. CHH, CAC and PWS performed all CT evaluations, designed the study, target delineations, and interpreted the study. CHH drafted the manuscript. YSK, LJL, KYH, LJW, SLL, CYC, SCL and YCL cared for the patients. YJC, LYW, and YPH gave advice on the work and performed the statistical analyses.

## Pre-publication history

The pre-publication history for this paper can be accessed here:

http://www.biomedcentral.com/1471-2407/11/37/prepub
